# Determinants of peer selection for collaborative group work of third-year bachelor students in a medical degree programme with learning communities

**DOI:** 10.1080/10872981.2022.2111743

**Published:** 2022-08-18

**Authors:** Yan Zhou, Jasperina Brouwer, Nicolaas Adrianus Bos, Agnes D. Diemers

**Affiliations:** a Center for Education Development and Research in Health Professions (CEDAR), LEARN, University Medical Center Groningen, University of Groningen, Groningen, The Netherlands; b Educational Technology, College of Teacher Education, Zhejiang Normal University, Zhejiang, China; c Educational Sciences, Faculty Behavioural and Social Sciences, University of Groningen, Groningen, The Netherlands

**Keywords:** Formal peer relationship, learning community, curriculum design, informal peer relationship, medical education

## Abstract

The social capital theory reveals the importance of peer relationships on students’ learning. However, it is unclear how students select their collaborators under the influence of their previous collaborations and backgrounds. This study explores to what extent students’ free selection choices for collaborators among their peers are based on previous collaboration in formally structured groups (i.e., learning communities (LCs)) and based on different students’ background characteristics. A parallel program was studied where students studied in one of four LCs for two years and after that, they have to find their own group members within or across LCs to finish their bachelor thesis in the third year. In total, 1152 students’ selections of their peers were analyzed. This paper presents the percentages of students choosing group members within or across LCs. It also considered the influence of students’ backgrounds, like sex, nationality, and academic performances on their peerchoices by logistic regression analysis. More than half of the students chose group members within their own LC, regardless of which LC they were in. Although the majority of the students chose collaborators within their own LC, still around 40% of students were willing to collaborate with others from different LCs with whom they had never collaborated before in the formal curriculum. Students’ backgrounds (i.e., sex, and academic performance) were also associated with their decisions. A high frequency of collaboration within formally structured groups enhances the students’ preference of group members from the same groups, but also informal peer relationships are crucial in students’ choices for collaboration. Students’ sex and academic performance influence their free choice of group members while nationality does not. Students with different academic levels have a higher chance to become group members when they collaborated before in formally structured groups than those students who had not had such a collaboration experience.

## Introduction

Many universities introduce collaborative learning, through learning communities (LCs) and other forms of small group teaching, in their curriculum design to improve the quality of education and students’ learning by fostering the formation of peer relationships [1–5]. Groups can be created formally (by the curriculum administration) or by free choice of the students themselves. Both can have beneficial effects on students’ learning, and relate to each other to some extent [6–9]. However, when students are free to create their own small groups later on in their educational degree programme (for example, for writing a bachelor thesis), it’s not clear how these earlier experiences affect their choices and what factors influence this peer selection process.

Peer relationships play a crucial role in student development and academic achievement[10]. As social capital theory indicates, social relationships are resources that can lead to the development and accumulation of human capital. Thus, social networks present social structures and resources to some extent. Besides, social capital may create common identity and shared understanding to bridge background differences by continuous interactions[11]. People connect with others so that they are able to establish trust relationships and access or mobilize resources through their social relationships [11,12]. These resources are embedded in the social networks. Thus, social capital refers to people accessing and using (borrowing or capturing) resources (i.e., information, wealth, power) to achieve their goals through social networks (based on their social relationships) [12–14]. Education consists of social settings so that people form their social relationships and exchange resources, which facilitate the formation of ‘bonding’ social capital within school [12,14,15]. The ‘bonding’ social capital within school indicates the ties between students[14]. In line with the social capital theory, students’ peer relationships are a source of emotional and academic support that might contribute to feelings of safety, companionship, and engagement. It can also enhance students’ adjustment to the learning environment, learning engagement, and achievement [16–19]. Two important mechanisms play a role in establishing peer relationships: homophily and propinquity [20–22]. Homophily relates to the tendency that students bond together with others who are similar to them, such as being of the same sex, ethnicity, or obtaining similar grades[5–8,23]. Propinquity is a form of homophily (location homophily)[24]. It presents the tendency for people to form friendships or other relationships with those whom they encounter often in the same place [20]. This implies that when students are involved in the same group and meet regularly, they have a higher chance to build relationships with each other.

When groups or organizations with a common goal interact productively, it benefits social capital formation [25]. Therefore, the establishment of LCs and small working groups in medical curricula fosters peer relationships formation, which is known to positively influence their collaboration and academic performances [16,26]. When students are formally assigned to a group for collaboration by the curriculum administration, it is expected that the students contact each other frequently and easily within a structured organization. Besides, by sharing their experiences in LCs, students might build both academic and social relationships [27]. This may facilitate the establishment of longitudinal support relationships and friendships with each other and may especially benefit first-year students in higher education [1,23,28–31]. This group-oriented practice extends the sense of community and enhances students’ collaboration within the same organizations [18,32,33].

Next to formal relationships that are created by the administration, the importance of informal relationships created outside of the formal curriculum has also been emphasized in recent years [7,34]. Informal relationships are known to be a source of motivation, accountability, support and well-being for students [35–37]. Students’ attitudes and motivation become similar with whom they are close within the informal network [7]. Notwithstanding the positive effects of informal relationship formation based on homophily, it may also lead to negative effects on learning, especially when the clustering takes place based on background and academic achievement. In international settings, for instance, it is known that both international and domestic students tend to form relationships mostly with peers of the same nationality [30,31], while the diversity of students’ backgrounds in classrooms or groups is known to benefit students’ academic performance [38]. Another negative side effect of homophily may be that low achievers have more problems building their own social groups because they seem to be excluded from the high achievers’ peer relationships to some extent [39]. Instead, they form peer relationships with other low-achievers and as such their academic achievements might not benefit from the high-achieving peers [39,40].

Research show that formal and informal peer relationships influence each other to some extent [33]. For instance, students may create relationships in their formally structured groups which they keep informally (out of class). Besides, students know others’ academic achievement levels or nationality by studying in a formally structured organization, and may therefore cluster their informal relationships with someone of the same nationality or performance [5].

Although various researches focus on formal and informal peer relationships on learning and achievement within higher education [34,36,40], it is not yet fully understood what the contribution is of such formal and informal peer relationships to students’ freely chosen small group formation at a later stage in medical education. Most research in higher education focuses on first-year students who are in a transition phase from secondary education to higher education [8,39,41]. Besides, little research has taken international medical undergraduate students into account. Thus, this study explores how students themselves choose their collaborators under the influence of both formal and informal relationships. This study is in the unique position to do so, because after two years of forming formal small groups within four separate LCs, students are free to choose their own groups within the curriculum at the beginning of the third year of their undergraduate (bachelor) medical program.

More insight into students’ relationships formation and what determines peer relationship formation may help curricula designers to enhance collaboration among students who have different backgrounds characteristics and academic performances.

So, the research questions of this study are:

1. Is there an association between students’ earlier formally structured groups (i.e., LCs) and collaborative group formation when students can freely select their own group members?
2. Is there an association between students’ background characteristics (i.e., age, sex, nationality, academic performance), and collaborative group formation when students can freely select their own group members?

## Methods

### Educational background

Since 2014, one medical school in the Netherlands adopted a new bachelor's curriculum based on four thematic learning communities (LCs): Sustainable Care (SC), Intramural Care (IC), Global Health (GH), and Molecular Medicine (MM) each consisting of approximately 100–120 students. These four LCs have their own theme, focuses, content, faculty and program design. LC SC focuses on long-term care, and epidemiological and clinical (first-line) research. LC IC deals with hospital and medical institutional care, and clinical and translational research. LC GH focuses on global healthcare issues, and epidemiological and socioeconomic research. LC MM deals with molecular and technological innovations, and translational and fundamental research. LC SC and LC IC are Dutch taught, and LC GH and LC MM are English taught. Before the start of the bachelor phase, students can freely choose and join one of the four LCs according to their interests of theme and language preferences. Due to their language ability, almost all international students join the two English LCs, Dutch students are free to choose one of the four LCs. After selection, students need to study in the LC of their choice for their entire three-year bachelor study program. It means students in one LC study together but they have no chance to study with students from other LCs in the formal curriculum.

In the first two years, to use the advantage of propinquity, the curriculum administration distributes students of the same LC randomly into formal small groups (ten students from the same LC in one small group) several times a year. It helps students to get to know each other and collaborate with peers within LCs and benefits their social capital formation. These groups were considered as formally established groups, since the LCs are formally organized.

Each small group is led by one master student. This master student helps to guide group discussion, information sharing, and materials learning. Students meet each other in those formal small groups twice a week. Each week one student serves as the team’s leader, guiding group discussion with the help of the master student. In the group meetings, students collaborate with group members to deal with some problems that relate to their learning materials. They also share their knowledge, experience and resources in groups. Students find and fill the gaps in their knowledge and competencies during the process. When students argue on some points of view during the process, the temporary team leader and master students record it and ask team members to find further information after the meeting, and discuss it in the next meeting by sharing what they have found.

In the third year, however, students have to organize bachelor thesis groups by themselves. In the bachelor thesis program, students spend one semester on their bachelor thesis and they need to work together with peers in small groups. Therefore, students need to organize small collaborative learning groups (normally around three to five people). Students are completely free in choosing their group members to collaborate in the bachelor thesis project, regardless of the LC they participated in during the previous two years. This bachelor thesis group formation starts at the end of the second year until the beginning of the second semester of the third year. Students have a few months to find and decide upon their group members and the subject of their bachelor thesis group. The bachelor thesis groups were considered as freely selected groups because students can freely choose their fellow students for collaboration. After selecting group members, students have a few weeks to discuss what they want to do for their bachelor thesis within their freely selected group. Students can come up with any topic they prefer regardless of their LC. Students also need to find a faculty as their supervisor and to discuss topics with their supervisors. Supervisors organize meetings with students and guide them to complete their thesis. All group members need to collaborate and finish their group thesis together. Students have to fill out a form at the end of the bachelor thesis program to clarify the process of their collaboration as well. Faculty scores students’ performance based on their thesis and their collaboration.

### Participants

All medical bachelor (BA) students of the year cohorts 2014–2015 (BA1415), 2015–2016 (BA1516) and 2016–2017 (BA1617) of the medical school in the Netherlands, who finished their bachelor thesis, participated in this study (n = 1152, 69% female, 31% male). Table 1 shows the demographic of students across four LCs. The percentage of female students in four LCs varied from 61% to 75%. The numbers of each cohort varied across the year (see Table 2). The mean age of all participants was 22.14 years (SD = 1.65). The majority of students were Dutch (N = 985; 86%), of which 615 (62%) chose one of the two Dutch taught LCs and 370 (38%) chose one of the two English taught LCs. Among the 167 international students (14%), five students (3%) chose a Dutch LC, whereas 162 (97%) chose an English LC. During the first two years of the bachelor, the composition of the small formal groups in the same LC changed randomly every semester for BA1415 and BA1516, and every half-semester for BA1617.

**Table 1.** Demographic of participants.

LC	Student number	Female		Domestic student	
SC	239	180	75.31%	235	98.33%
IC	381	273	71.65%	378	99.21%
GH	280	192	68.57%	185	66.07%
MM	252	154	61.11%	179	71.03%
**Total**	**1152**	**799**	**69.36%**	**977**	**84.81%**

**Table 2.** Student choice of mixed or mono group by cohort.

Cohort	Student number	Mono group		Mixed group	
BA1415	394	211	53.55%	183	46.45%
BA1516	376	201	53.32%	175	46.42%
BA1617	382	239	62.47%	143	37.53%
**Total**	**1152**	**651**	**56.51%**	**501**	**43.49%**

### Measurements

#### Students’ free choice of group formation

Based on students’ LCs, this study created binary group types as ‘mono group’ and ‘mixed group’, as the outcome and the dependent variable of this study. A ‘bachelor thesis’ group consisting of students from the same LC was called a ‘mono group’, whereas a ‘bachelor thesis’ group with students from different LCs was described as a ‘mixed group’.

All analyses were performed for group characteristics and personal characteristics, known to be associated with the students’ team selections. Group characteristics included: (1) students’ LCs and (2) if students collaborated with at least one group member in the past two years in formally structured small group learning.

#### Student background characteristics

Personal characteristics included age, sex, nationality and their average written test scores in the first two years. Students were separated into domestic students and international students according to students’ nationality. Written tests are curriculum-dependent tests and assess students’ medical knowledge four or five times every semester. The results of the written test are combined as one semester test which scores range from 0 to 10. This study used the average score of students’ first two years (four semesters) written tests score (before bachelor thesis group selection) to present the level of students’ academic performance. The average of students’ written test scores varied from 4.50 to 8.84.

Since previous studies found that low-performing students have difficulties contacting high-performing students or getting support from high-performing students [8], this study explored the collaboration between high and low achievers when students can freely select their group members. Based on the distribution of the written test, this study classified the students into three groups: The top 25% of students were classified as high achievers (score ≥ 7.01), and the middle 50% of students were classified as medium achievers (score > 6.17 and <7.01), and the bottom 25% of students as low achievers (score ≤ 6.17).

### Data collection

Students’ group selection results were obtained from the administration, who is responsible for the organization of the bachelor thesis program. Students’ background information, such as age, sex, nationality, and previous academic achievements (written test scores) were collected from the administration office of the Medical Faculty. All data were integrated into one dataset, according to students’ numbers. To anonymize the data, the students’ numbers were encrypted to research codes. Thus, individual data cannot be traced back to the individual without the file of encryption. Only the main researcher (YZ) was able to access the original data.

### Statistical analyses

First, descriptive analyses were performed to show the result of students’ freely selected group formation based on two types of groups, considering their backgrounds. These descriptive data showed how do students’ formally structured groups and backgrounds related to the students’ freely chosen small group formation. Homophily was represented by similarity regarding students’ background characteristics (i.e., age, sex, nationality, and written test scores) and attending the same LC. This study explored if students’ background characteristics were associated with their collaborative group formation when students can freely select their own group members. Meanwhile, propinquity was represented by previous collaboration in this study, which can be seen as meeting at the same place and time. Therefore, this study explored if students’ earlier formally structured groups associated with their collaborative group formation when students can freely select their own group members. Since the continuous data ‘written test score’ was non-normally distributed, this study performed the Mann-Whitney U test to compare students’ academic performance between different types of groups. The Chi-square test was performed to explore if students’ group decisions differed among LCs.

Second, this study analyzed the correlation between independent variables (age, sex, nationality, LC, previous collaboration, academic performance) by Pearson correlation coefficient (used for analyzing between continuous variables: age and written test score) and Kendall correlation coefficient (used for analyzing between continuous variables and categories variables).

Third, the Box-Tidwell method was used to verify the linearity to the logit for the continuous independent variables in logistic regression analysis to perform binary logistic regression. [42] Other nominal categorical variables (such as sex, nationality) were dichotomized.

Fourth, to evaluate what factors relate to students’ group decisions (i.e., mix group or mono group), this study did a binary logistic regression analysis, creating a final model that only included predictions with *p* < 0.05. Odds Ratios (OR) and 95% confidence intervals (CI) were calculated. The SPSS 26.0 program was used for all statistical analyses [43].

### Ethical approval

The study was approved by the Ethical Review Board of the Netherlands Association of Medical Education (NVMO), dossier number 2019.4.8. The data were directly obtained from the administration and anonymized and confidentially treated, in line with the ethical guidelines.

## Results

### Group selection result

The total of 1152 participating students formed 316 small groups by their free choice. In the three aforementioned cohorts, 130 mixed bachelor thesis groups existed (i.e., consisting of students from different LCs) and 186 mono bachelor thesis groups existed (i.e., consisting of students from one LC). Table 2 shows the result of students’ group selection. The majority of students chose group members from their own LC (56.51%) (Table 2). Cohorts BA1415 and BA1516 had a similar result of students’ choices (around 53% of students chose a mono group), whereas BA1617 had a higher percentage of students (62%) who chose a mono group. It is of note that the number of times that formal small group composition changed in BA1617 (eight times) was twice as frequent as in cohort BA1415 and BA1516 (four times), which means that the number of fellow students in cohort BA1617 that students collaborated with in formally structured groups was twice as much as for BA1415 and BA1516 students. The previous collaboration in formally structured groups also positively related to students’ choices of staying within their own LC. The percentage of students who collaborated before with at least one of their group members was higher in the mono group (71%) than in the mixed group (54%) (Table 3).

**Table 3.** Student choice of mixed or mono group by student background and previous collaboration.

variables	Mono group (n = 651)	Mixed group (n = 501)
Age, mean (SD)	22.09 (1.649)	22.23 (1.724)
Female, n (%)	474 (72.81)	325 (64.87)
Domestic, n (%)	567 (87.10)	418 (83.43)
Collaborated before, n (%)	462(70.97)	275 (54.89)
Written test score, mean (SD)	6.68 (.60)	6.57 (.61)
High achiever, n (%)	184 (28.26)	104 (20.76)
Medium achiever, n (%)	325 (49.92)	253 (50.50)
Low achiever, n (%)	142 (21.81)	144 (28.74)
LC, n (%)		
SC	138 (21.20)	101 (20.16)
IC	209 (32.10)	172 (34.33)
GH	158 (24.27)	122 (24.35)
MM	146 (22.43)	106 (21.16)

The percentage of students from the four LCs who chose a mono or a mixed group were similar to each other. In all four LCs, between 55% and 58% of the students chose a mono group (Table 3). There is no significant difference between mono of mixed group choice among the different LCs (*X^2^* (3) = .901, *p* = .825). So, in general, when students had a free choice, they were more likely to form groups with students with whom they collaborated before within the same LC, regardless of which LC they were involved in.

### Choices across Dutch and English language LCs

Out of the 130 mixed groups, 66 groups consisted of students from LCs with the same language (so only English or Dutch taught LCs) of which 25 groups (37.88%, all from the English taught LCs) contained international students. Of the 64 mixed groups that contained students from both English and Dutch taught LCs, 52 groups (81.25%) were formed by domestic students only. The majority of mixed groups (67.57%) that contain international students consisted of students only from the English taught LCs. So it means that when students can freely select their team members and formed mixed groups, international students from English LCs had little collaboration with Dutch students from Dutch LCs, but Dutch students (from English LCs as well) preferred to collaborate with other Dutch students from Dutch LCs.

### Factors related to students’ freely selected group formation

Next, background characteristics (sex, nationality) and academic performance were related to the choice of the students for a mixed or a mono group. Students who chose a mono group were compared with those who preferred a mixed group composition per background characteristic. In the different cohorts, the percentage of female students varied between 67% and 72%, with a total average of 69%. The proportion of female students in the mono group was 73%, and in the mixed groups this was 65%. This means that more female students preferred to choose mono groups whereas more male students preferred mixed groups (35% over 27%). Concerning nationality, the proportion of domestic students compared to international students in mono groups was higher (87%) than that in the mixed groups (83%). In other words, domestic students have a slight preference for mono groups over mixed groups whereas international students favor mixed groups. Regarding students’ academic performance, the percentage of high achievers was higher in the mono groups (28%) than in the mixed groups (21%). While, the percentage of low achievers was the other way around, so lower in the mono groups (22%) than in the mixed groups (29%). The percentage of medium achievers was similar in both groups (around 50%). Additionally, the average score of students’ written test scores was significantly higher (*p* < .001) in the mono groups. This is in line with the finding that more high- and less low achievers were present in the mono groups than in the mixed groups. Table 4 shows the students’ groups selection according to group members’ academic performances. Of the total of 316 groups, only 65 groups (21%) consisted of both higher and lower achievers (Table 4), of which 74% concerned the mono groups. The majority of groups (79%) were formed by students with similar academic performance.

**Table 4.** Student choice of mixed or mono group by academic performance.

Academic performance in groups	Mono group	Mixed group
Groups contain both high and low achievers	48	17
Groups do not contain both high and low achievers	138	113

A bivariate correlation analysis was performed between the different background characteristics and the choice of the students to be in a mono group or in a mixed group. Table 5 shows the correlation relationships between every two independent variables. All correlation coefficients between two independent variables were between −0.8 and 0.8 (Table 5), meaning that the influence of multicollinearity can be ignored. Two continuous independent variables (age and written test score) had a linear relationship with the dependent variable (group decision) based on the logit transformation values (*p* = .929 > .05 and *p* = .189 > .05). Thus, the two continuous independent variables can be included in the binary logistic regression analysis without dichotomizing it.

**Table 5.** Correlation analysis of demographic and performance characteristics.

	LC	gender	nationality	collaborated	Written test score	age
LC	1					
gender	.091**	1				
nationality	.332**	.141**	1			
collaborated	−.061*	−.064*	.026	1		
Written test	−.014	−.088**	−.048*	−.013	1	
age	.074**	.144**	.293**	−.047	−.084**	1

*The Kendall and Pearson rank correlation is significant at the 0.05 level (2-tailed).

**The Kendall and Pearson rank correlation is significant at the 0.01 level (2-tailed).

### Binary logistic regression analysis result

Table 6 shows the three variables that had a significant influence on students’ group selection and that were kept in the equation: sex, earlier collaboration and written test score. The odds (95% CI) of female students choosing a mono group was 1.312 times higher than male students. This means that female students were more likely to choose mono groups than male students. When the written test score increased by one unit (for example, from 6 to 7 on a scale from 1–10), the odds of students choosing a mono group increased 1.347 times, which means that students with higher written test scores were more likely to choose a mono group compared to students with lower written test scores. If students collaborated with at least one group member, the odds of students organizing a mono group increased 2.012 times, which means that students who collaborated with other students in formally structured groups in the previous two years more often chose group members from the same LCs than students who had never collaborated in formally structured groups before.

**Table 6.** Binary logistic regression analysis of student characteristics and choice of a mono group.

Variables	B	S.E	Wald	df	Sig.	Exp(B)	95% C.I. for Exp(B)	
							Lower	Upper
Sex	.272	.133	4.150	1	.042	1.312	1.010	1.704
Collaborated	.699	.128	29.709	1	.000	2.012	1.565	2.588
Written test	.298	.103	8.395	1	.004	1.347	1.101	1.648
Constant	−2.338	.690	11.489	1	.001	.097		

## Discussion and conclusions

This study explored to what extent students’ experience in previous formally structured groups (i.e., LCs) related to the formation of freely selected groups in a later stage of their bachelor study and what factors related to students’ choices (i.e., bachelor thesis group selection). The result showed that the majority of the students chose collaborators within their own LC. Students’ previous collaboration experience, sex and academic performance were associated with students’ freely selected group formation.

First, more than half of the students chose group members within their own LC, regardless of which LC they were in or the language of the LC. This shows that when students met each other frequently at the same place and time, they connected to each other, which is consistent with the propinquity or proximity effect as considered in previous studies [21,33,39,44,45]. In addition, the number of times students collaborated before in formally structured groups related to their peer selection decision: The more frequently they collaborated with peers within their own LC already, the more they favor choosing group members within their own LC. When students interacted more frequently with each other in a formal setting, i.e., in the same LC, they got to know each other better and this created a preference to choose these fellow students freely for the bachelor thesis project. This is consistent with other studies that students prefer to collaborate with others with whom they collaborated before but with a precondition that only higher familiarity with each other increases the chance of collaboration [39,46,47]. This means that in order to enhance the function of formally structured groups, faculty should consider offering students more possibilities to become familiar with each other rather than just putting them randomly in groups. This is in line with previous research that LCs create a safe learning environment where students establish peer relationships [48].

Although the majority of the students chose collaborators within their own LC, still around 40% of students were willing to collaborate with others from different LCs with whom they had never collaborated before in the formal curriculum. This can also be considered as an advantage because it creates more diversity in the bachelor thesis groups, which may enhance creativity [49]. The selection across LCs indicates that students’ informal peer relationships (i.e., friendships) may positively contribute to students’ freely selected collaborator decisions [50]. Since these informal relationships are not controlled by faculty, curriculum designers need to be aware of peer selection mechanisms and the role of formally structured groups in the curriculum [26]. On one hand, even though faculty provide formally structured groups, it does not mean students will collaborate with their group members when they can freely choose each other. On the other hand, when a faculty strives for more diversity in small groups, most students will remain in their own niche. Moreover, since students’ formal and informal peer relationships influence each other to some extent [33], faculty may consider how to affect students’ informal peer relationships by revising their formal curriculum design depending on the aims the curriculum has.

Second, students’ backgrounds were related to how they choose their group members as well. Sex seems to play a role since this study found that female students were more likely to choose collaborators within LCs compared to male students. Another influencing background factor on students’ free group choices may be nationality. Although this study found that nationality did not influence students’ decisions of the mixed or mono groups, the international students in English LCs seemed to have less connections with domestic students out of class than domestic students even in the same LCs. The number of mixed groups containing international students formed by students from English LCs alone was twice as high as mixed groups formed by students from LCs with different languages. The language barrier may limit international students’ collaboration with domestic students to some extent [51]. Even though international students in medical school in the Netherlands need to learn Dutch before they start their clinical rotations in their master's study, most of them are still unable to communicate in Dutch within their bachelor's program.

Finally, academic performance could also play a role when students have a free choice in group composition. High achievers preferred to select collaborators within their own LC while low achievers were more likely to choose collaborators from different LCs. Medium achievers did not show a preference for collaborators from either their own or other LCs. This result is in line with previous findings that high achievers may have more connections with other students in the formally structured organization, especially with other high achievers [8,39]. Thus, when students can freely select their collaborators, high achievers might have more options to choose peers from their own LC. As a result, low achievers seem to be excluded from the high achievers’ networks to some extent, offering them limited options to choose from within their own LC [8]. That might explain the finding that low achievers chose to collaborate with students from other LCs. This is in line with previous research [39,41].

Furthermore, the results of this study identified that the collaboration between high and low achievers was much less frequent than collaboration between students with other levels of achievement (for example, between medium and low achievers, or between high and medium achievers). It is consistent with other studies that low achievers have little chance to collaborate with high achievers [8,39]. In this study, only 21% of the groups contained both high and low achievers. In these groups, the majority of groups (74%) were formed by students from the same LC. It is likely that the formally structured group organization, such as LCs, improves the familiarity and interpersonal relationships between high achievers and low achievers in such a way that propinquity increases the chances to collaborate together.

### Strengths and limitations for study

This study not only reveals to what extent experiences in formally structured group organizations relate to the free choice of collaborative groups but also how informal relationships influence this free selection of collaboration. As far as we know, this is the first study that explores how formal and informal peer relationships influence medical undergraduate students to form their freely chosen collaborative group. This study provides a better understanding of the factors that play a crucial role in peer selection processes when students can create freely selected groups. The findings seem quite robust since this study makes use of three cohorts with more than 1100 students who selected their peers for the bachelor thesis project. Furthermore, since this study included international students, the difference in group formation between international students and domestic students could be compared.

This study has some methodological limitations. This study only considered some objective background characteristics, while other subjective students’ attributes, like prosocial attitude, cultural difference, learning strategies and motivation, and language proficiency, may also play a role in students’ choices [16,34,41,52]. Future research should take more control variables, such as students’ subjective characteristics, into account to more deeply understand the students’ peer relationships formation based upon the interaction of all of these factors. For instance, language proficiency may affect students’ peer relationships formation, especially the relationships between domestic and international students [52]. Domestic students who lack English proficiency may have difficulty connecting with international students. International students who lack Dutch proficiency may also have difficulty integrating into domestic academic groups. Thus, future research could evaluate students’ language proficiency (both Dutch and English) and treat it as a control variable. This study did not consider the subject of the bachelor thesis as a background characteristic. Since the choice of the group composition happened before the choice of the subject of the thesis, it is possible that the thesis subjects are less likely to play a direct role in their choice of group members. However, it would be interesting to study the relationships between the theme of the LCs and the subject of the bachelor thesis in future research. More ecological studies that take cultural differences into account may be needed in the future as well [53]. Moreover, future studies could consider to analyze additional control variables. For example, it could be suggested in future research to analyze process data on students’ collaboration in bachelor thesis programs as well. In this way, faculty can figure out how students collaborate with each other when they work in the teams made by themselves, and how it differs from when they were in the groups organized by faculty.

No longitudinal qualitative data on students’ informal relationships were collected in this study. The effect of formally structured groups on students’ peer relationships may fade over time [54]. In the future it will be useful to analyze different types of students’ peer relationships with social network research to consider how these relationships develop over time by social network analysis methods [49]. This may add to the existing knowledge of how students form and develop different types of informal relationships and how informal and formal relationships influence each other over time and affect students’ academic performance.

### Implication for practice

This study offers some useful insights for curriculum designers. In the light of the findings of this study, curriculum designers are advised to use formally structured groups for students since it helps them to form and keep collaborative relationships in and out of class. This might be useful when students get to know each other. Later on in the study program enhancing diversity might be more important [49]. When this is the aim of the curriculum, it is recommended to enhance the diversity of populations in small groups as much as possible rather than randomly distributing international and domestic students to foster formal interactions between international and domestic students. The diversity of students’ backgrounds in small groups can create conditions that are conducive to active thinking and intellectual engagement, which can potentially improve the quality of the study group, and thereby their academic performance [6,49].

Besides, to improve informal collaboration among different levels of academic achievers, this study would suggest offering more chances of collaboration in formally structured groups to students among different levels of academic performance in order to enhance the collaboration between different levels of academic achievers. If curriculum developers want to help international students to make full use of all possibilities to collaborate, it would be useful to consider students’ language ability and offer them some additional language support when needed [55]. In addition, even though the effect of formally structured groups may fade over time, it is still necessary for curriculum designers to design ongoing activities to help students’ interactions to increase the probability of broader students’ academic connections during their study [6,54].

Furthermore, earlier collaborations between LCs in the first two years may enhance the interactions between international students and domestic students. This will benefit both students from Dutch as well as English LCs and foster better use of the available diversity.

### Conclusion

This study reveals that formally structured groups enhance the possibility of students’ collaborative relationship formation. Students’ attributes, like sex and academic performances, influence students’ freely selected group formation. A high frequency of collaboration within formally structured groups enhances the students’ preference for group members from the same community. Finally, it is important to notice that students’ personal relationships out of class may also play an important role in students’ choices for collaboration.

## Data Availability

The data that support the findings of this study are available from the corresponding author, N.A. Bos, upon reasonable request.
